# A Pilot Study of miRNA Expression Profile as a Liquid Biopsy for Full-Marathon Participants

**DOI:** 10.3390/sports9100134

**Published:** 2021-09-24

**Authors:** Tomoaki Kuji, Takehito Sugasawa, Shin-ichiro Fujita, Seiko Ono, Yasushi Kawakami, Kazuhiro Takekoshi

**Affiliations:** 1 Doctoral Program in Sports Medicine, Graduate School of Comprehensive Human Sciences, University of Tsukuba, 1-1-1 Tennodai, Tsukuba 305-8577, Japan; s1930394@s.tsukuba.ac.jp or; 2 Research and Development Division, Blue Industries Inc., ArcaCentral Bldg. 14F, 1-2-1 Kinshi, Sumida, Tokyo 130-0013, Japan; 3 Laboratory of Laboratory-Sports Medicine, Division of Clinical Medicine, Faculty of Medicine, University of Tsukuba, 1-1-1 Tennodai, Tsukuba 305-8577, Japan; take0716@krf.biglobe.ne.jp (T.S.); shin.ichiro.fujita.03@gmail.com (S.-i.F.); y-kawa@md.tsukuba.ac.jp (Y.K.); 4 Master’s Program in Medical Sciences, Graduate School of Comprehensive Human Sciences, University of Tsukuba, 1-1-1 Tennodai, Tsukuba 305-8577, Japan; s2021387@s.tsukuba.ac.jp

**Keywords:** small RNA, miRNA, exosome, full marathon, RNA sequencing, NGS

## Abstract

Exosomal microRNA (miRNA) in plasma and urine has attracted attention as a novel diagnostic tool for pathological conditions. However, the mechanisms of miRNA dynamics in the exercise physiology field are not well understood in terms of monitoring sports performance. This pilot study aimed to reveal the miRNA dynamics in urine and plasma of full-marathon participants. Plasma and urine samples were collected from 26 marathon participants before, immediately after, 2 h after, and one day after a full marathon. The samples were pooled, and exosomal miRNAs were extracted and analyzed using next-generation sequencing. We determined that the exosomal miRNA expression profile changed under time dependency in full marathon. New uncharacterized exosomal miRNAs such as hsa-miR-582-3p and hsa-miR-199a-3p could be potential biomarkers reflecting physical stress of full marathon in plasma and urine. In addition, some muscle miRNAs in plasma and urine have supported the utility for monitoring physical stress. Furthermore, some inflammation-related exosomal miRNAs were useful only in plasma. These results suggest that these exosomal miRNAs in plasma and/or urine are highly sensitive biomarkers for physical stress in full marathons. Thus, our findings may yield valuable insights into exercise physiology.

## 1. Introduction

The marathon is a long-distance running event in which athletes compete for position and time on a 42.195 km public road course [1]. The event is based on an ancient Greek legend and began as a new track-and-field event to establish the first modern Olympics [1]. Visualization of marathon exercise intensity is essential for conditioning and physical condition management of athletes, and there is, thus, a need for the identification of characteristic biomarkers [2].

“Liquid biopsy” has emerged as a diagnostic method to include molecular biomarkers detected in the blood or other bodily fluids such as urine or cerebrospinal fluid of patients [3]. Blood, among other such bodily fluids, includes cancer origin molecules that allow for molecular characterization of the disease, facilitating early diagnosis and monitoring, prognosis, and personalized medicine [4]. The molecular contents of nucleic acid released from cancer are circulating tumor cells, circulating tumor DNA/RNA (ctDNA/RNA), cell-free DNA/RNA (cfDNA/RNA), RNAs, and protein and are released as extracellular vesicles, such as exosomes, into body liquids [5,6,7]. In particular, miRNAs have been spotlighted as potential biomarkers for clinical diagnosis [8,9]. MiRNA is a class of non-coding RNA and has an approximate length of 21–25 nucleotides. In addition, it has a function of post-transcriptional expression of genes in eukaryotes [10]. miRNA-mediated transcriptional repression plays an essential role in a wide range of biological processes such as development, cell proliferation, differentiation, apoptosis, and metabolism [10]. miRNA is released from its transcribed genome into the bloodstream [4,11]. Such miRNA contained in bodily fluids can also be detected in healthy people and may change naturally through physical activity [11]. Indeed, previous studies have shown that intense exercise changes blood miRNA expression, suggesting that miRNA may be a suitable biomarker of physical stress [4,11,12,13,14]. In addition, miRNAs in urine were studied as biomarkers in a previous study and can be considered as less invasive biomarkers for visualizing marathon exercise intensity [15]. In particular, exosomal miRNA is contained in nano-sized extracellular vesicles and released from cells and transferred between cells [16]. In previous studies, exosomal miRNA has been shown to be expressed under exercise [17]. However, miRNA biomarkers for the assessment of competitive sports activity have not been established. Thus, this study aims to reveal the exosomal miRNA dynamics in urine and plasma of full-marathon participants by using next-generation sequencing (NGS).

Our previous study showed that the absolute amount of cfDNA increases during a full marathon [18]. In previous studies, miRNAs have been reported to increase in blood after exercise [19]. However, only blood was analyzed in those studies. In addition, mainly muscle-specific miRNAs have been analyzed. Based on these previous studies, we considered that if we could comprehensively analyze miRNAs in both blood and urine and discover common biomarkers for them, we could reduce the cost of analysis, find physiological commonalities in body fluid circulation, and provide a new perspective on sports physiology. Here, as a continuation of our research activities, we focus on miRNA as a biomarker screening study during a full marathon as a high-intensity exercise.

In this pilot study, we analyzed the expression pattern of exosomal miRNAs in plasma and urine before and after a full marathon. The samples were pooled together for analysis to protect personal information and to examine the expression levels of exosomal miRNA in plasma and/or urine. We found that the exosomal miRNA expression patterns also differed time dependently in plasma and/or urine and could be optimized for use in the bodily fluid types as biomarkers for monitoring physical intensity under the full marathon. Thus, our pilot study may offer insights toward the development of an evaluation tool for improving sports performance.

## 2. Materials and Methods

### 2.1. Ethical Approval and Study Overview

**Figure 1:**
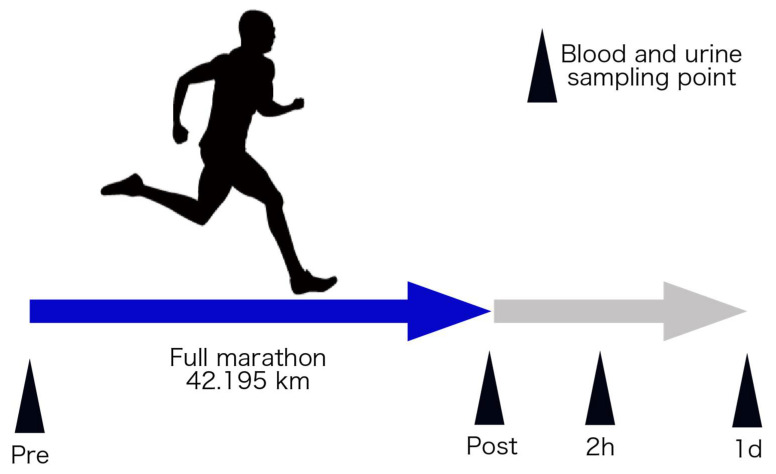
Schematic overview of the experimental protocol. Pre, immediately before the full marathon; post, immediately after the full marathon; 2 h, two hours after full marathon; and 1 d, 1 day after full marathon.

An overview of this study’s protocol is shown in Figure 1. This study was approved by the Ethics Committee of the Faculty of Medicine at the University of Tsukuba (Approval number: 274). All subjects received an explanation and documents in advance regarding the experiment’s purpose, content, and safety issues and indicated their informed consent.

### 2.2. Study Participants

A total of 26 healthy male subjects who engaged in aerobic exercise at least twice per week were recruited for the study. Their mean age was 25.2 years, with a standard deviation (SD) of 7.3; mean height was 172.0 cm (SD 5.3); and mean weight was 64.9 kg (SD 6.9). Blood pressure diastolic/systolic was 125.7/74.7 mmHg (SD 9.1/7.9), and mean marathon time was 3 h 27 min 14 s (SD 58 min 48 s). All subjects completed the 38th Tsukuba Marathon, and they determined their own pace. The subjects were instructed not to drink alcohol, to obtain sufficient sleep, and to avoid overeating before the marathon. On the day of the full marathon, the participants freely performed warm-up exercises and drank water. Notably, the subjects were the same as the participants in one of our previous papers [18,20]. The current study focuses on exsomal miRNAs in plasma and urine, and it includes new knowledge that was not found in the previous paper [18,20]. In this study, three subjects were removed after exceeding the criteria for maximum levels of general biomarkers, including C-reactive protein and urinary albumin, in sedentary conditions before the full marathon [20]. The final number of samples for analysis was 23.

### 2.3. Sample Collection

Outside air conditions on the day of the full marathon were 12.7 °C and a relative humidity level of 60.6%. Blood samples of the participants were collected into EDTA blood collection tubes at four time points: immediately before the marathon (Pre; also before warmup), immediately after the marathon (Post), two hours after the marathon (2 h), and one day after the marathon (1 d). The participants were instructed to drink only water between the Post and 2 h collection points. The collected blood samples were centrifuged at 3000 rpm for 15 min at 4 °C. Aliquots of the plasma were then dispensed into 1.5 mL microtubes and stored at −80 °C until further analysis. These descriptions about the sample collection are the same as that reported in our previous study [18,20].

### 2.4. Extracted Exosomal RNA in Plasma and Urine

Exosomal RNA in plasma sample (total pooled volume 4 mL) was extracted using a Plasma/Serum Circulating and Exosomal RNA Purification Kit (NORGEN BIOTEK, Thorold, ON, Canada). Exosomal RNA in urine samples (total pooled volume 30 mL) was extracted using a Urine Exosome Purification Maxi Kit (NORGEN BIOTEK, Thorold, ON, Canada). After this, RNA purity check, library preparation, sequencing, and data analysis were outsourced to Novogen (Beijing, China). RNA purity was then checked using a NanoPhotometer (IMPLEN, Munich, Germany), and RNA concentration was measured using Qubit RNA Assay Kit in Qubit 2.0 Fluorometer (Thermo Fisher Scientific, Waltham, MA, USA). RNA integrity was assessed by using the RNA Nano 6000 Assay Kit of the Agilent Bioanalyzer 2100 system (Agilent Technologies, Santa Clara, CA, USA).

### 2.5. Library Preparation for Small RNA Sequencing

Small RNA sequencing was performed in Novogene Biotech (Beijing, China) as outsourcing. The methods including library preparation and data analysis are described as the following, according to the reports produced by the Novogene Biotech. An amount of 3 μg of total RNA per sample was used as input material for the small RNA library. Sequence libraries were generated using the NEBNext multiplex small RNA library prep set for Illumina (New England Biolabs, Ipswich, MA, USA), and index codes were added to attribute a sequence for each sample. Briefly, NEB 3′SR adapters were directly and specifically ligated to the 3′ ends of exosomal miRNAs, siRNAs, and piRNAs. After the 3′ ligation reaction, the SRRT primers hybridized to the excess 3′SR adapters (which remained free after the 3′ ligation reaction) and converted the single-stranded DNA adapters into double-stranded DNA molecules in order to prevent adapter dimer formation. In addition, as dsDNA is not a substrate for ligation mediated by T4 RNA ligase 1, it does not ligate to the 5′SR adaptor in the subsequent ligation step. We ligated the 5′ ends adaptor to the 5′ ends of miRNAs, siRNAs, and piRNAs. Next, first-strand cDNA was synthesized using M-MuLV reverse transcriptase. PCR amplification was performed using LongAmp Taq 2X master mix, SR primers for illumina sequencing platform, and index primers. PCR products were purified in an 8% polyacrylamide gel (100 V, 80 min). DNA fragments corresponding to 140–160 bp (the length of small non-coding RNA plus 3′ and 5′ adapters) were collected and dissolved in 8 μL of elution buffer. Finally, the quality of the libraries was assessed on an Bioanalyzer 2100 system (Agilent Technologies, Inc., Santa Clara, CA, USA) using DNA High Sensitivity Chips (Agilent Technologies). Raw data for RNA sequencing were acquired using a high-throughput sequencing system, NovaSeq 6000 (Illumina, San Diego, CA, USA).

### 2.6. Data Analysis Process

**Figure 2:**
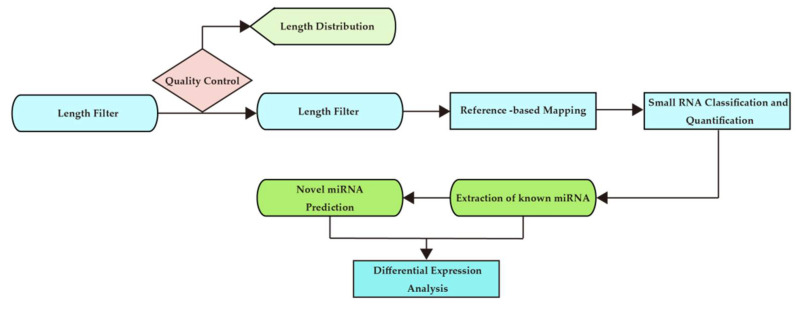
Bioinformatics analysis workflow chart.

The analysis process is shown in Figure 2.

Raw data (raw reads) and clean data (clean reads) in quality control FASTQ format were obtained by removing the following reads from the raw data: those that contained ploy-N, 5′ adapter contaminants, or ploy A, T, G, or C; those that did not contain 3′ adapters or insertion tags; and those that were of low quality (Table S1). Simultaneously, the Q20, Q30, and GC contents of the raw data were calculated (Table S2). Next, a specific range of lengths from the clean reads was selected to perform all downstream analyses (Table S3).

The small RNAs were mapped to a reference (GRCh38) using Bowtie (version 0.12.9) and analyzed for expression and distribution without mismatch [21].

The mapped small RNA tags were used to find known miRNAs. Using miRBase20.0 as a reference, modified software miRDeep2 (version 2_0_0_5), ViennaRNA (version 2.1.1) and sRNA-tools-cli were used to retrieve potential miRNAs and analyze secondary structure [22]. In addition, miRNA counts and base biases were obtained for the first position of identified miRNAs of a specific length and for each position of all identified miRNAs, respectively.

To remove tags derived from protein coding genes, repetitive sequences and small RNA tags were mapped from the specified species itself to RepeatMasker (version open-4.0.3) and Rfam databases.

Features of the hairpin structure of miRNA precursors can be used to predict novel miRNAs. The available software miREvo (version 1.1) and miRDeep2 were integrated to predict novel miRNAs by exploring the secondary structure, dicer cleavage sites, and minimum free energy of small RNA tags not annotated in the previous step [22,23]. At the same time, we obtained the identified miRNA counts and the base bias for the first position of a particular length and for each position of all identified miRNAs, respectively (Figure S1).

In the previous alignments and annotations, some small RNA tags were mapped to multiple categories. To map all unique small RNAs to only one annotation, the priority rule of known miRNA > rRNA > tRNA > snRNA > snoRNA > repeat > gene > NAT-siRNA > gene > novel miRNA > ta-siRNA was followed. The total rRNA ratio was used as a marker as a sample quality indicator.

The expression level of miRNAs was estimated by TPM (transcripts per million) using the following criteria normalization formula: normalized expression = mapped read counts/total reads × 1,000,000 [24].

Differential expression analysis used the DEGseq (version 2010) R package [25]. The *p*-value was adjusted using *q*-value [26]. *Q*-value was set as the threshold for significant differential expression by default.

### 2.7. Selection of miRNA Potential Biomarkers by Filtering with Sufficient Expression and Fold Change

In order to narrow down the miRNA biomarker candidates for monitoring the physical activity intensity of the full-marathon participants, the following process was employed using the miRNA expression profile. The expression data were filtered with a read count of 100 in time-point samples collected immediately after the full marathon in plasma and urine, respectively. Then, we added 1 to the zero read count of exosomal miRNA in the urine and plasma samples before the full marathon. The fold change was then calculated between the TPM values of the Pre time-point sample and the Post time-point sample to yield the fold change value. miRNAs with a fold change of more than 2 in urine and blood were selected as biomarker candidates.

## 3. Results

### 3.1. Quality Information of the NGS Run and Informatics Analysis

Regarding RNA data from plasma and urine, we obtained a total of 19–36 million reads (Table S1). In terms of quality, Q20 was over 93% and Q30 was over 89% (Table S1), and 7–31 million reads (Table S2) were clean reads. The total quantity of RNAs was 0.9–7 million (Table S3). Among these data, there were 0.4–1.3 million known miRNAs (Table S3). In addition, there were 175–580 novel miRNAs in plasma (Table S3), and there were 976–2982 novel miRNAs in urine (Table S3).

Previous studies have shown that miRNAs as new biomarkers can be found in plasma with around 0.5 million reads [27]. In the present study, since the average number of total reads in plasma is about 2.8 million, we expected that miRNAs as novel biomarkers could be found. The software miRDeep2 was used in this analysis. Compared with previous studies, our results have a Spearman’s correlation of more than 0.85 between different miRNA algorithms [27]. From this comparison, the number of reads required to find known and novel miRNAs in plasma has been reached, and the quality of the data was considered to be at a certain level. Thus, we proceeded with our analysis based on these data.

### 3.2. MiRNA Expression Profile during Full Marathon

**Figure 3:**
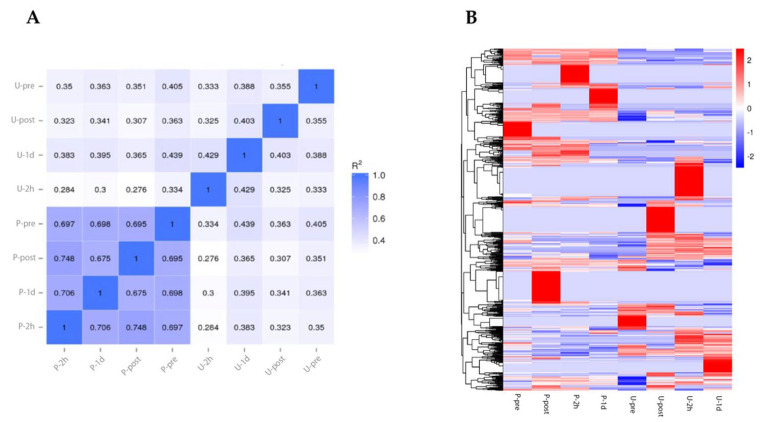
miRNA expression profile under full marathon. (**A**): Pearson correlation between samples. (**B**): Heatmap showing DEMs. Abbreviations: P-pre (plasma sample before marathon), P-post (plasma sample immediately after marathon), P-2h (plasma sample after two hours of marathon), P-1d (plasma sample after one day of marathon), U-pre (urine sample before marathon), U-post (urine sample immediately after marathon), U-2h (urine sample two hours after marathon), and U-1d (urine sample one day after marathon).

Visualization of marathon exercise intensity is important for conditioning and management of the physical condition of athletes, and there has been a need to determine characteristic biomarkers for such visualization. To identify such biomarker candidates, we performed miRNA-seq analysis. We measured the samples at pre-exercise, during exercise, and post-exercise time points in urine and blood. The miRNA profile showed greater correlation between time points in blood samples than in urine (Figure 3A). Next, differential expression miRNA (DEM) analysis identified 878 miRNAs. These RNA expressions were changed explicitly at certain time points (Figure 3B). These results suggest that miRNA expression changes in a time-dependent manner in a full marathon.

**Figure 4:**
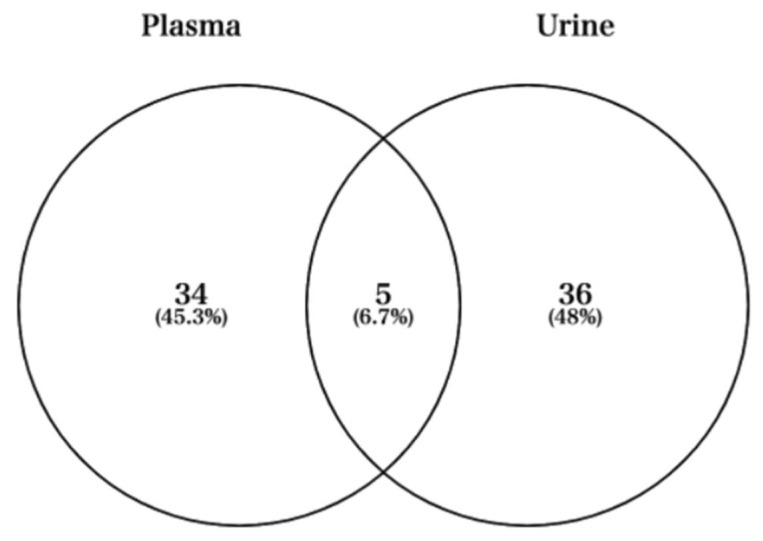
Venn diagram showing the fold changes cut-off 2 between Post and Pre time-points in plasma and urine.

**Figure 5:**
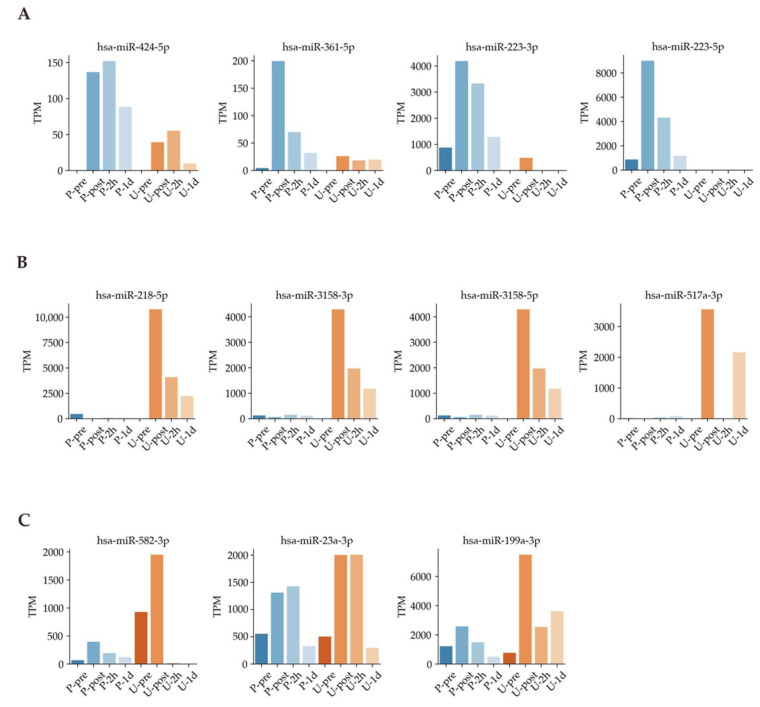
Exercise-dependent miRNA expression patterns. (**A**): Time dependence in plasma samples. (**B**): Time dependence in urine samples. (**C**): Time dependence in both plasma and urine samples.

Next, we used Venn diagrams to select miRNAs with sufficient expression and high fold changes as biomarkers (Figure 4). For example, miRNAs such as hsa-miR-424-5p, hsa-miR-361-5p, hsa-miR-223-3p, and hsa-miR-223-5p were upregulated in plasma (Figure 5A). In addition, other miRNAs such as hsa-miR-218-5p, hsa-miR-3158-3p, hsa-miR-3158-5p, and hsa-miR-517a-3p were upregulated in urine (Figure 5B). Moreover, miRNAs such as hsa-miR-582-3p, hsa-miR-23a-3p, and hsa-miR-199a-3p were upregulated in both plasma and urine (Figure 5C). Table 1 shows comparisons of these miRNAs and previous studies. The previous studies reported that these miRNAs reflect cancer, inflammation, or muscle damage. Moreover, novel miRNAs, for which there were no reports on characterization, were identified (Table 1). These results suggest that miRNAs that are unique and/or common in bodily fluids could serve as candidates for biomarkers.

### 3.3. Timeline of Known Muscle-Specific MiRNA Expression Patterns in Plasma and Urine

**Figure 6:**
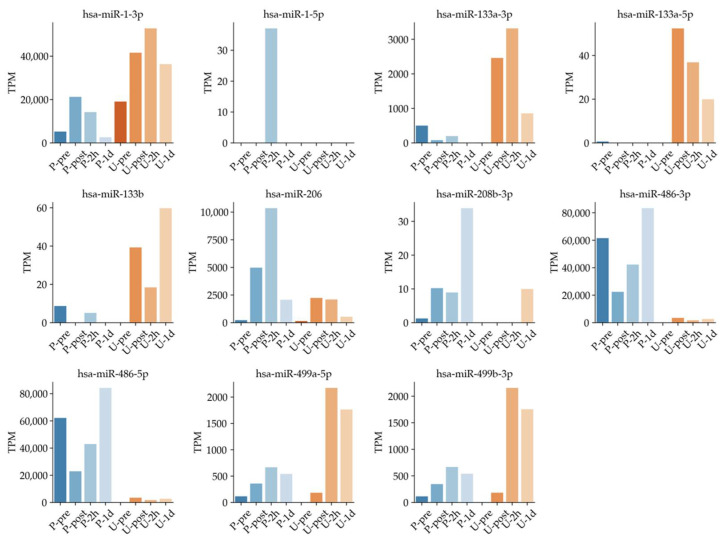
Known muscle-specific miRNA expression showing time dependence in plasma and urine samples.

Previous studies suggested that miRNA expressions were regulated by exercise [4,17,19,49]. In particular, circulating muscle-specific miRNAs (known as myomiRs) could reflect muscle status such as damage, exercise, and recovery [4,9,17,19]. However, none of those studies time dependently compared the exosomal miRNAs in plasma and urine collected from the same samples. Known muscle-specific exosomal miRNA changes were confirmed in the overlapped region in plasma and urine (Figure 6). For example, has-miR-1-3p in plasma increased rapidly immediately after full marathon and decreased with time. On the other hand, has-miR-1-3p in urine peaked at the U-2h time point, then tended to decrease over time. These expression patterns were also confirmed in hsa-miR-499a-5p and hsa-miR-499-3p. These results are in line with expression patterns of miRNAs in urine, along with expressions of miRNA in plasma, reported in past studies [14,19,41,50,51].

## 4. Discussion

In order to monitor and improve athletic performance, more detailed monitoring of physical function requires simple and rapid biomarkers [2]. Previous studies have reported that common blood indicators such as K+, BUN, creatinine, CK, and LDH can be monitored for athlete performance [52,53]. However, given recent advances in genome analysis technology, it may be possible to establish a more precise assessment method. Since liquid biopsy was developed as a diagnostic method to use molecular biomarkers in the blood or other bodily fluids [4,17,54], we focused on nucleic acids and studied the variation before and after a full marathon. For example, a previous study showed that the absolute amount of cfDNA in plasma is elevated during a full marathon [18]. Since miRNAs, such as those included in exosome, also have attracted attention as diagnostic biomarkers for the systemic response of exercise performance [4,11,14,17,19], we sought to identify biomarker candidates to visualize physical activity by analyzing plasma and urine samples from full-marathon participants using NGS in this pilot study.

Our results showed higher correlations between plasma samples than between urine samples (Figure 3A). A total of 878 DEMs were identified to respond under a full marathon (Figure 3B). Interestingly, our results suggested that some miRNAs changed in a time-dependent and bodily fluid-dependent manner (Figure 5 and Figure 6 and Table 1). For example, we found that hsa-miR-424-5p, hsa-miR-361-5p, hsa-miR-223-3p, and hsa-miR-223-5p increased specifically in plasma (Figure 5A and Table 1). In particular, hsa-miR-424-5p, hsa-miR-223-3p, and hsa-miR-223-5p are reported as inflammation-related miRNAs [12,55,56,57,58]. However, previous research has not investigated the expression of has-miR-223-3p and hsa-miR-223-5p in urine during a full marathon. We confirmed that the expression levels of has-miR-223-3p and hsa-miR-223-5p were upregulated in Post time-point in plasma during a full marathon (Figure 5A). Furthermore, hsa-miR-424-5p was also upregulated only in plasma after the full marathon in our filtering (Figure 5A), and similar trends were observed in previous studies [19,41]. In previous studies, this miRNA was not upregulated in 10 km and half-marathon and was only found to be expressed in the full marathon [41]. This suggests that it could be only expressed in cases of prolonged intense exercise or long-term running. These results suggest that these miRNAs can be useful only in plasma for monitoring physical stress during full marathon.

Next, we found that exosomal miRNAs such as hsa-miR-218-5p, hsa-miR-3158-3p, hsa-miR-3158-5p, and hsa-miR-517a-5p specifically increased in urine (Figure 5B and Table 1). For example, hsa-miR-218-5p is related to chronic obstructive pulmonary disease (COPD), which is a risk factor for cancer [38]. Specifically, the expression of hsa-miR-218-5p is high in healthy people, while it is low in smokers and people with COPD [38]. Therefore, it is presumed that the expression level of hsa-miR-218-5p is increased by activation of lung function. Furthermore, we found two miRNAs, namely hsa-miR-3158-3p and hsa-miR-3158-5p, in urine samples, and their functions have not been clearly determined in previous research for full marathons. Thus, these results indicate that cancer-related miRNAs and unknown-function miRNAs could be useful for monitoring physical activity using urine samples.

Next, we selected overlapped miRNAs in a Venn diagram for both urine and plasma (Figure 4 and Table 1). Then, hsa-miR-1-3p, hsa-miR-206, hsa-miR-23a-3p, hsa-miR-582-3p, and hsa-miR-199a-3p were upregulated in plasma and urine (Table 1). Hsa-miR-582-3p expression correlated with the overall and recurrence-free survival of non-small-cell lung cancer patients and activating effect on Wnt/β-catenin signaling for stem cell pluripotency regulation and cell fate decisions during development [45]. The expression of hsa-miR-582-3p may be due to the activation of respiratory and pulmonary functions under exercise and the differentiation of stem cells and cells in the lung. Hsa-miR199a-3p has been reported as an miRNA biomarker for colorectal cancer (CRC) in serum, and the expression was significantly higher in CRC patients than in non-cancer patients [47]. Although mRNAs such as hsa-miR-582-3p and hsa-miR-199a-3p have been reported in previous studies in oncology, there have been no previous reports on their expression under exercise in healthy individuals [45,47]. Hsa-miR-23a-3p has been widely reported in previous exercise studies [43,44,59,60,61,62]. In our study, we focused on plasma and urine to confirm the upregulation of hsa-miR-23a-3p. In addition, it is reported that for participants subjected to resistance exercise, hsa-miR-23a-3p was upregulated in the group that ingested protein and in a group that was not detected in previous research [44]. Furthermore, previous studies in postmenopausal women have reported that miR-23a-3p upregulation levels also correlate with TRAP5b, a gene involved in bone resorption [62]. Moreover, previous research using human renal cell carcinoma cells showed that upregulation of hsa-miR-23a-3p increases cell survival, proliferation, and migration and has been reported to be useful as a biomarker [61]. In contrast to the above previous research, hsa-miR-23a-3p has been reported to be downregulated due to high-intensity exercise such as resistance exercise by muscle biopsy [43]. It has also been reported in previous studies that it is involved in the regulation of myogenic differentiation by repressing the expression of fast myosin heavy chain isoforms [60]. In addition, downregulation of hsa-miR-23a-3p after exercise has also been reported, but these may be due to differences in sampling, such as muscle biopsy [43,59]. This discrepancy in tissue and blood studies under exercise could be explained by the opposite phenomenon in tissue and plasma miRNA expression by drug-induced Injury in previous research [63].

Other miRNAs in Table 1 such as hsa-miR-1-3p and hsa-miR-206 are reported as muscle specific miRNAs. These miRNAs have been suggested to be useful as biomarkers for analyzing exercise intensity [14,19,42]. They could be useful for monitoring physical intensity under full marathon in both plasma and urine. Thus, we further investigated muscle-specific miRNAs comprehensively in plasma, such as has-miR-1-3p, hsa-miR-1-5p, miR-206, and miR-208b-3p (Figure 6). Expression levels of has-miR-1-3p, has-miR-206, has-miR-208b-3p, has-miR-499a-5p, and has-miR-499b-3p tended to increase earlier in blood after exercise and later in urine (Figure 6). Our results support the utility of several previously reported circulating miRNAs, and we observed their new dynamics in plasma and urine.

For example, in a different study using the same sample, Figure 4 of the paper shows that leukocytes and myoglobin changed several tens of times after exercise [18]. By contrast, Figure 5B of the present miRNA study shows a maximum of about 10,000-fold change, and the TPM change in Figure 6 shows an increase in several thousand-fold change. Therefore, one of the features of the present study is that it shows the possibility of the use of these miRNAs as highly sensitive biomarkers compared with the existing kinetic markers.

Variations of existing exercise markers, myoglobin in blood and white blood cells, have been shown in our previous research [18]. The trends in myoglobin and leukocytes, rising and falling before and after the full marathon, are similar to the fluctuation pattern of muscle-specific miRNAs such as miR-1-3p, miR-133a-5p, miR-206, miR499a-5p, and miR-499b-3p in plasma and urine in the present study. Based on the above findings, along with the variation of general hematological measurements such as leukocyte and myoglobin, we confirmed that some inflammation-related and muscle-specific miRNA showed higher fold changes than the general markers, suggesting the possibility of more sensitive markers. These results suggested correlations between the miRNAs and muscle damage or inflammation markers. However, because this study used pooled samples, statistical correlation analysis could not be achieved.

This study has several limitations. First, a limited number of samples were collected from full-marathon runners. Many of the participants were men; thus, gender bias was present. Second, a next-generation sequence sample was created from a pooled sample to protect personal information because pooled samples were used for anonymization of personal information as a method approved by the Ethical Review Committee. In addition, well-established and accepted methods such as restricting access to data to essential study personnel, coding subjects by number or another method of de-identifying data, password protecting hard drives and computers, and using physical security such as locked storage facilities had to be overcome. Thus, we were not looking at variations in the whole but rather the pooled miRNA expression level. Third, the discrepancy between the existing studies and this study on miRNA expression may be due to differences in sampling. For example, our results showed that TPM data of hsa-miR-133a-3p, hsa-miR-133a-5p, and hsa-miR-133b were missing (Figure 6). In terms of data, the number of sequence reads in plasma in this study is 19–36 million, which is higher than in previous research [64]. Another factor that could be considered as a hypothesis is the issue of sampling [63]. This is because we collected samples outdoors and used pooled samples. On the other hand, Russell and Aaron P. et al. used muscle biopsy [59]. Other studies that analyzed the dynamics of miRNAs in plasma before and after exercise used procedures different from the RNA extraction method used in this study and different centrifugation times and speeds, which may have affected our results [51,65]. This is one of the issues to consider going forward, since such minor differences in extraction methods may also have an impact on the RNA data obtained. Method of collection and other factors may have affected the state of the miRNAs, causing them to be degraded. A comprehensive analysis of general biochemical analysis and cfDNA, in addition to miRNAs, can compensate for missing data and different sampling. In past studies, cfDNA has been analyzed in some cases, and we believe that analyzing the data in combination with it will provide physiological findings from other perspectives [18]. The findings and their implications should be discussed in the broadest context possible. The reason only male subjects were collected this time was that 80% of the marathon participants were male, and it was difficult to collect female subjects. For future studies, one improvement would be to obtain a larger number of samples in a larger competition in order to balance the gender ratio, to standardize the sampling, and to analyze the samples separately. In addition, we suggest comparing miRNA expression changes in plasma and urine of runners who completed the marathon quickly and those who completed it at a slower pace and conducting a detailed comparative study on exercise load for detailed analysis. Thus, we suggest establishing and improving sampling and data standardization to perform the best timing for collecting subjects in future studies.

## 5. Conclusions

The aim of this study was to illuminate exosomal miRNA dynamics in both urine and plasma in full-marathon participants using NGS. We found that the exosomal miRNA profile changed significantly in a time-dependent manner. Moreover, potential new biomarkers, such as hsa-miR-582-3p and hsa-miR-199a-3p, for determining physical stress levels were found in both urine and plasma of the marathon participants. While research protocols such as sampling limited the scope of this pilot study, it can serve as a roadmap for future multi-sampling and time dynamics studies in sports physiology.

## Figures and Tables

**Table 1:** Example of exercise-dependent miRNA list from Figure 4.

Type of RNA	Example of Characteristic	Sample with Expression	Reference
hsa-miR-424-5p	Pancreatic cancer, muscle, and inflammation	Plasma	[12,28,29]
hsa-miR-361-5p	Lung cancer and muscle (smooth muscle)	Plasma	[30,31]
hsa-miR-223-3p	Colon cancer, muscle, and inflammation	Plasma	[12,32,33]
hsa-miR-223-5p	Malignant neoplasms including vulvar carcinoma, non-small cell lung cancer, bladder cancer, prostate cancer, and inflammation	Plasma	[12,34,35,36,37]
hsa-miR-218-5p	Chronic obstructive pulmonary disease and muscle (smooth muscle)	Urine	[38,39]
hsa-miR-3158-3p	Unknown	Urine	
hsa-miR-3158-5p	Unknown	Urine	
hsa-miR-517a-3p	Lung cancer and inflammation	Urine	[12,40]
hsa-miR-1-3p	Muscle specific	Plasma and Urine	[4,19,41]
hsa-miR-206	Muscle specific	Plasma and Urine	[4,19,42]
hsa-miR-23a-3p	Muscle	Plasma and Urine	[43,44]
hsa-miR-582-3p	Lung cancer and muscle (smooth muscle)	Plasma and Urine	[45,46]
hsa-miR-199a-3p	Colorectal cancer and muscle	Plasma and Urine	[47,48]

## Data Availability

The next-generation sequencing data presented in this study are not available because it includes personal information.

## Supplementary Materials

The following are available online at https://www.mdpi.com/article/10.3390/sports9100134/s1, Table S1: Quality summary of sequence read data, Table S2: Data filtering summary, Table S3: Type, quantity of total RNA and percentage, Figure S1: Sequencing distribution of total RNA.
