# Supplementary material for: A Pilot Study of miRNA Expression Profile as a Liquid Biopsy for Full-Marathon Participants
Source: Sports (Basel). 2021 Sep 24;9(10):134. doi: 10.3390/sports9100134 (PMC8539081; doi:10.3390/sports9100134)
Supplement: Supplementary file 1 [file sports-09-00134-s001.zip › sports-1351406-supplementary.pdf]

Table S1. Quality summary of sequence read data.

Sample	Reads	Bases	Error Rate	Q20	Q30	GC content
P-pre	26,700,063	1.335 G	0.01%	95.95%	92.75%	52.87%
P-post	30,186,552	1.509 G	0.01%	96.20%	93.40%	52.33%
P-2h	27,394,864	1.370 G	0.02%	93.39%	89.92%	50.52%
P-1d	28,410,157	1.421 G	0.02%	94.30%	91.01%	50.19%
U-pre	19,103,375	0.955 G	0.01%	97.08%	94.41%	54.20%
U-post	36,654,940	1.833 G	0.01%	96.27%	92.95%	53.24%
U-2h	33,698,200	1.685 G	0.01%	96.07%	92.84%	54.23%
U-1d	34,899,738	1.745 G	0.01%	96.17%	92.82%	54.97%

Abbreviations: Sample means Sample id. Reads means statistics of the original sequence data. Bases means sequence number multiplied by the length of the sequence, expressed in giga base pairs (Gbp). Error rate means Sequencing error rate. Q20 means Phred values greater than 20 base percentage accounted for the overall base. Q30 means Phred values greater than 30 base percentage accounted for the overall base. GC content means The G base and C base accounted for the overall base.

Table S2. Data filtering summary.

Sample	Total Reads	N% > 10%	Low Quality	5' Adapter contamination	3′ Adapter or Insert Missing	With Poly A/T/G/C /	Clean Reads
P-pre	26,700,063 (100.00%)	14 (0.00%)	329,307 (1.23%)	6,295,834 (23.58%)	4,183,299 (15.67%)	10,874 (0.04%)	15,880,735 (59.48%)
P-post	30,186,552 (100.00%)	147 (0.00%)	433,909 (1.44%)	23,358,870 (7.74%)	2,756,135 (9.13%)	33,307 (0.11%)	24,627,184 (81.58%)
P-2h	27,394,864 (100.00%)	104 (0.00 %)	959,160 (3.50%)	4,338,166 (15.84%)	13,042,915 (47.61%)	8006 (0.03%)	9,046,513 (33.02%)
P-1d	28,410,157 (100.00%)	103 (0.00%)	775,318 (2.73%)	4,855,849 (17.09%)	15,099,673 (53.15%)	10,184 (0.04%)	7,669,030 (26.99%)
U-pre	19,103,375 (100.00%)	88 (0.00%)	142,368 (0.75%)	198,891 (1.04%)	2,183,578 (11.43 %)	38,126 (0.20%)	16,540,324 (86.58%)
U-post	36,654,940 (100.00%)	9 (0.00%)	296,482 (0.81%)	431,934 (1.18%)	4,829,196 (13.17%)	92,331 (0.25%)	31,004,988 (84.59%)
U-2h	33,698,200 (100.00%)	15 (0.00%)	395,809 (1.17%)	545,835 (1.59%)	5,212,985 (15.47%)	69,513 (0.21%)	27,484,043 (81.56%)
U-1d	34,899,738 (100.00%)	15 (0.00%)	337,739 (0.97%)	327,173 (0.94%)	4,513,274 (12.93%)	54,835 (0.16%)	29,666,702 (85.01%)

Abbreviations: Sample means Sample ID. Total Reads mean total sequenced reads. N% > 10% means percentage of reads with N > 10%. Low Quality means percentage of low-quality reads. 5′ Adapter Contamination means percentage of reads with 5′adapter contamination. 3′ Adapter or Insert Missing means percentage of reads without 3′ adapter or insert. With PloyA/T/G/C means percentage of reads with PloyA/T/G/C tails. Clean Reads means total clean reads followed by clean reads as a percentage of raw reads.

Table S3. Type, quantity of total RNA and percentage.

Type	P-pre	P-pre (%)	P-post	P-post (%)	P-2h	P-2h (%)	P-1d	P-1d (%)
Total RNA	2,714,180	100.00%	7,378,428	100.00%	1,950,701	100.00%	956,359	100.00%
Known RNA	1,325,211	48.83%	679,705	9.21%	665,899	34.14%	439,583	45.96%
Novel RNA	457	0.02%	580	0.01%	257	0.01%	175	0.02%

Type	U-pre	U-pre (%)	U-post	U-post %)	U-2h	U-2h (%)	U-1d	U-1d (%)
Total RNA	1,597,905	100.00%	4,717,808	100.00%	2,830,579	100.00%	2,148,286	100.00%
Known RNA	90,402	5.66%	70,931	1.50%	50,283	1.78%	88,936	4.14%
Novel RNA	976	0.06%	2740	0.06%	2982	0.11%	2047	0.10%

Abbreviations: Total RNA means the quantity of sRNA reads mapped to genome. Known miRNA means the number and percentage of sRNAs reads mapped to known miRNA. Novel miRNA means the number and percentage of sRNAs reads mapped to novel miRNA.

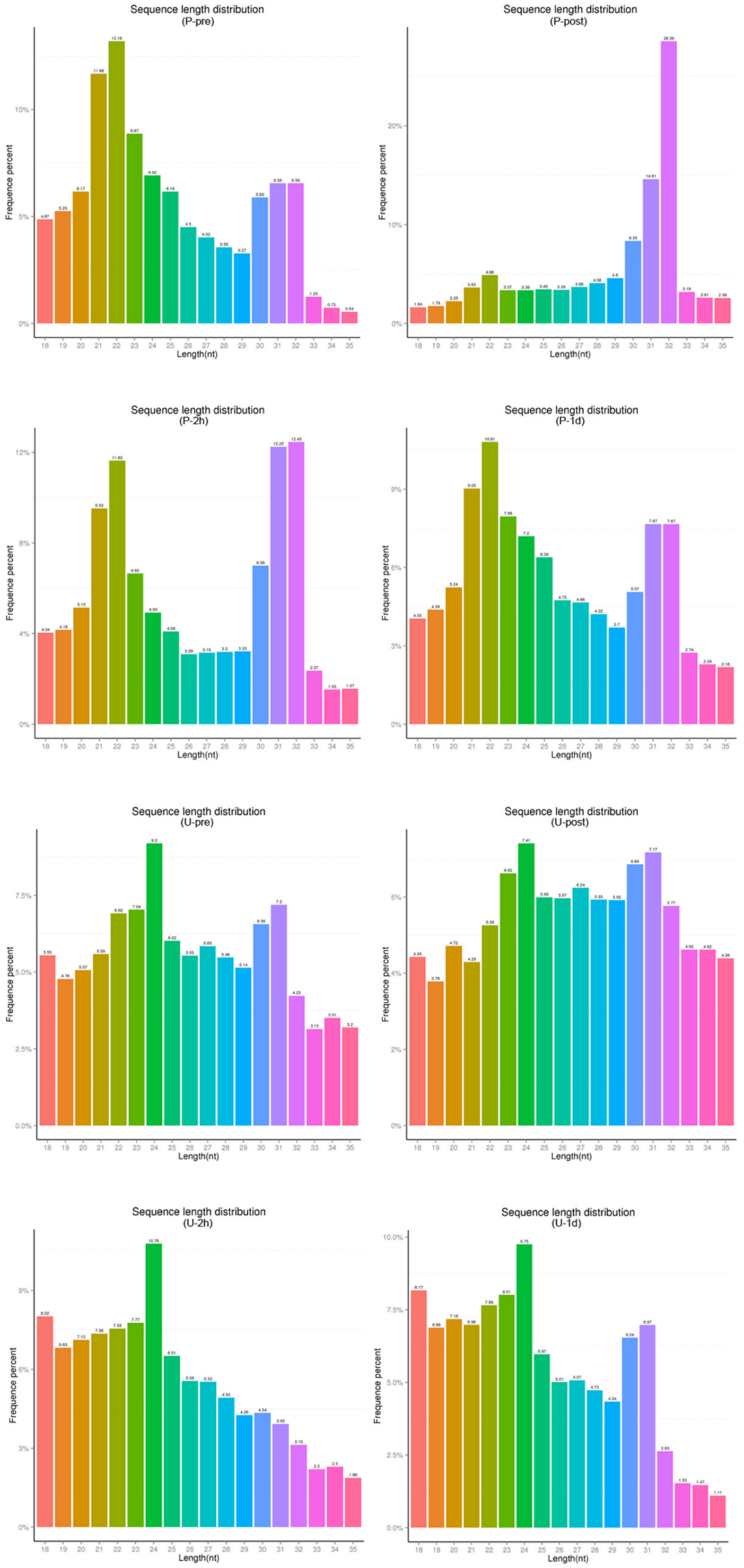

Figure S1. Sequencing distribution of total RNA.

Abbreviations: X-axis means sRNA length (nt). Y-axis means frequency percentage (%)
